# Bending Energy Schemes for Discrete‐Spring‐Network Structural Modelling of Red Blood Cells

**DOI:** 10.1002/cnm.70114

**Published:** 2025-11-19

**Authors:** Osayomwanbor Ehi‐Egharevba, Mingzhu Chen, Fergal J. Boyle

**Affiliations:** ^1^ School of Mechanical Engineering Technological University Dublin Dublin Ireland

**Keywords:** bending energy, discocytes, echinocytes, red blood cells, stomatocytes, vesicles

## Abstract

Red blood cells (RBCs) undergo large structural deformation, including bending, when passing through capillaries. They also exhibit a range of complex shapes such as stomatocytes, discocytes and echinocytes that form due to altered blood pH and salt levels, ingested drugs and adenosine triphosphate depletion. Discrete‐spring‐network structural models of RBCs employ different numerical treatments of the continuum bending energy. This affects bending accuracy and the prediction of accurate RBC shapes. This research compares three representations called bending energy scheme (BES) A, B and C to evaluate their accuracy in shape predictions. BES A, seen throughout the literature, is based on the formulations of Kantor and Nelson, while BES B and BES C are, respectively, spring‐based and node‐based curvature calculation methods based on the formulations of Jülicher. Flat and enclosed spring‐network membrane test cases are presented, and predictions using the schemes are compared. The flat membrane test cases explored the bending of stiff and soft membranes while the enclosed membrane test cases evaluated equilibrium vesicle and RBC shape prediction, including predictions of the stomatocyte‐to‐discocyte‐to‐echinocyte sequence. Predictions showed that BES A and BES B have limitations and can underestimate the true bending deformation. Additionally, BES A and BES B are also unable to capture the necking behaviour critical to the accurate prediction of complex RBC shapes. BES C on the other hand was seen to be accurate and robust and predicted shapes closely matched expected biological shapes. Based on this research, BES C is recommended for all future spring‐network RBC structural modelling.

## Graphical abstract

This research investigated three bending energy schemes for discrete‐spring‐network structural modelling termed bending energy scheme (BES) A, B and C. Flat and enclosed membrane test cases were presented, and predictions using the schemes were compared. All schemes accurately predicted the bending deformation of stiff membranes; however, for soft membranes, like vesicles and red blood cells, BES C proved to be the most accurate and robust, while the commonly employed scheme BES A was found to underestimate the true bending deformation.

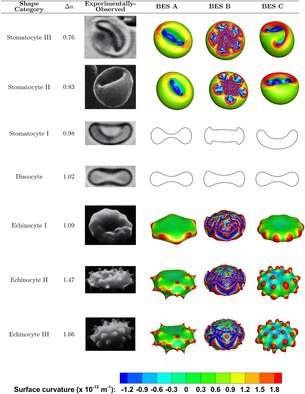

## 1 Introduction

**FIGURE 1:**
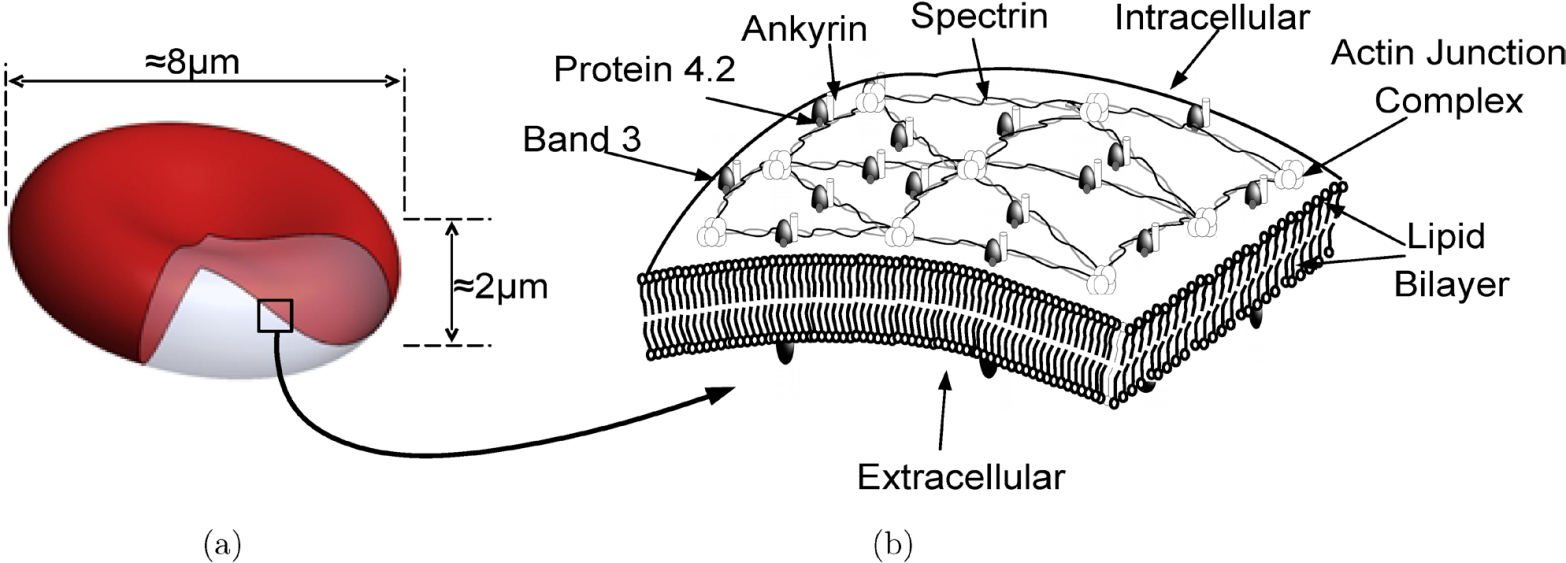
Schematic diagrams of a healthy RBC and its structure. (a) A healthy discocytic RBC with a diameter of 8 μm and a thickness of 2 μm and (b) the composite RBC wall is formed by the PM attached to the cytoskeleton. The PM is composed of lipid molecules with hydrophilic heads and hydrophobic tails to form a lipid bilayer. The cytoskeleton is an elastic mesh‐like network composed of many proteins.

Red blood cells (RBCs) are highly deformable enucleated biconcave‐shaped discs approximately 8 μm in diameter and 2 μm in thickness. The structure of a RBC is composite in nature. The RBC wall is composed of a plasma membrane (PM) attached to an underlying cytoskeleton. This composite wall encloses a liquid cytosol which is composed of haemoglobin and enzymes. The PM is mainly made of phospholipids, like phosphatidylcholine and sphingomyelin, in equimolar amounts with unesterified (free) cholesterol [1, 2]. The lipid molecules, each with a hydrophilic head and a hydrophobic tail, are arranged into two leaflets to form a bilayer. This structural orientation of the bilayer prevents surface area dilation while providing a low resistance to out‐of‐plane bending and virtually no resistance to in‐plane shearing. The cytoskeleton, which is an elastic mesh‐like network composed of proteins like spectrin, actin, protein 4.1 and ankyrin, confers shear resistance to the RBC wall (see Figure 1).

RBCs are very small and delicate and, as such, experimental studies to understand the fluid dynamics in the microcirculatory network are difficult; numerical models have been developed as a viable alternative to study RBCs in this situation. Current numerical models of RBC structural mechanics can be categorised as either continuum‐based or discrete‐based. Continuum models treat the RBC wall as a thin elastic sheet where the model energy is defined through strain–deformation relationships. These models have been successfully employed in numerical blood‐flow simulations [3, 4, 5] to model flow‐induced RBC deformations. However, they are not suited to capturing intrinsic RBC behaviour like thermal fluctuations. Discrete models on the other hand are mathematically simpler than continuum models and model a RBC as a network of springs and nodes [6, 7, 8, 9, 10, 11]. They have been used to study flow‐induced RBC deformation [12, 13, 14] and can capture thermal fluctuations on the cell surface [15] which may affect cell–cell and cell–vessel wall interactions. Discrete RBC models have also been used to simulate diseased RBCs [16, 17].

**FIGURE 2:**
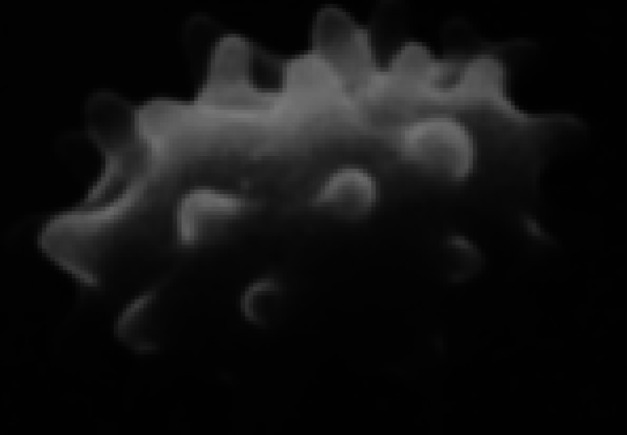
An echinocytic RBC. The cell is spiculated with small spikes evenly spread over its surface. The equilibrium shape is heavily influenced by the bending mechanics. Reprinted with permission from Bessis [19].

Modelling of complex RBC shape morphologies like stomatocytes and echinocytes is also an important area of increasing interest. Stomatocytes are RBCs with a single concave indentation, and arise from normal discocytes under conditions such as low pH and salt levels, cationic amphipathic drugs and cholesterol depletion [18]. Their presence may also indicate genetic disorders such as hereditary stomatocytosis. Echinocytes (see Figure 2), on the other hand, are RBCs with about 10–50 small spikes evenly spread around the surface of the cell. In vivo, echinocytes are not formed by erythropoiesis, i.e., the creation of RBCs from stem cells, but form due to high pH and salt levels, anionic amphipathic drugs, adenosine triphosphate depletion and cholesterol addition [18]; they may also be attributed to the following diseased states: bleeding peptic ulcers, low‐potassium RBCs and liver disease [20]. While continuum‐based RBC models have been successfully used to numerically study echinocytic RBCs [21, 22, 23, 24], discrete models have solely been applied to simple RBC shapes such as discocytes and stomatocytes. This had been the case until the numerical studies of Chen and Boyle [25] and Geekiyanage et al. [26] where hybrid models were used to produce the full stomatocyte‐to‐discocyte‐to‐echinocyte sequence. These complex shapes are heavily influenced by the bending mechanics which compete with shear resistance under energy minimisation to derive the shape topology. The hybrid models employed in the prediction of echinocytes [25, 26] differ from conventional discrete models by utilising discrete mechanics for all other components of the RBC combined with a discretised‐continuum description for the bending mechanics. This is in contrast to conventional discrete models that use a discrete‐based definition for all membrane mechanics.

Aside from the various equilibrium shapes of RBCs, micro‐haemodynamics studies have also shown a rich array of shapes and dynamics in flow. RBCs in shear flow tumble at low shear rates [27, 28] while tank treading as ellipsoidal shapes at high shear rates [29, 30]. In capillary flows, RBCs are also seen to form parachute‐like shapes [7]. Interesting phenomena like RBC aggregation [31], cell–cell, cell–vessel wall and cell–plasma interactions further signify the complex physics that has to be predicted by numerical models. Discrete models capture this physics through shearing and bending energy minimisation while conserving RBC surface area and volume and can be validated by performing a variety of test cases. The validity of RBC shear elasticity is investigated by comparing predictions from the numerically simulated optical tweezers experiment against experimental measurements [32]. RBC bending elasticity on the other hand is validated by comparing the numerically predicted equilibrium RBC shape to the cross‐sectional RBC shape of Evans and Fung [33] derived from experimental measurements. This predicted shape is achieved by the volume deflation of a sphere with the same surface area as a RBC [34]. While this bending validation test case can show qualitative agreement with Evans and Fung [33], quantitative validation of numerical bending elasticity is difficult to ascertain; however, the role of cytoskeleton contributions to equilibrium shapes cannot be decoupled and is still under discussion [35].

An efficient and accurate discretisation for the numerical prediction of the membrane bending energy is an important issue in the numerical studies of vesicles and cells. Conventional discrete spring‐network models have not been previously used to predict complex RBC morphologies due to the discretisation of the bending energy employed in these models. In this work, three different treatments of the bending energy, seen in the literature for discrete‐spring‐network models are investigated; they differ based on the calculation of curvature and thus the bending energy. The first discretisation, referred to here as BES A (bending energy scheme A), is based on the work of Kantor and Nelson [36] and is the most commonly seen discretisation in the literature. BES B (bending energy scheme B) is a spring‐based bending scheme adapted from the curvature definition proposed by Jülicher [37] and it is explicitly shown in this work that BES B is equivalent to BES A under certain conditions. Lastly, BES C (bending energy scheme C) employs a direct calculation of the curvature at the spring nodes as proposed by Jülicher [37]. BES A, BES B and BES C have been previously compared for spherical capsules [38]; their relative performance has not been compared for complex RBC shapes until this work. For the first time, through a variety of flat and enclosed spring‐network membrane test cases, it is shown that BES A and BES B have limitations and might underestimate the true bending deformation. Critically, the test cases presented here highlight that BES C should be used in future RBC structural modelling.

## 2 Materials and Methods

### 2.1 Bending Mechanics

Biomembrane bending mechanics are typically described by four models: Canham [39], Helfrich [40], bilayer‐couple [41] and area difference elasticity (ADE) [42]. All of these bending models are based on the curvature of a surface. At any point on a surface, the curvature is defined as the inverse radius of a circle which passes through that point and follows the topology of the surface [43]. As the circle at the point can be defined in multiple directions, the maximum and minimum curvatures are defined as $C_{1}$ and $C_{2}$, respectively.

The first model to describe the bending energy of a biomembrane was formulated by Canham. This model, sometimes referred to as the minimal model [44], was developed to explain the biconcave shape of a RBC. The curvature of both leaflets of the PM is assumed to be equal and the bending energy is given by

(1) $$E_{\text{Canham}}=\frac{k_{c}}{2}\int C^{2}\mathrm{dA}$$

where $k_{c}$ is the physical bending modulus, the curvature $C=C_{1}+C_{2}$, dA is the area of a surface element, and the integration is over the entire membrane surface.

Helfrich [40] extended the Canham model to account for the possible chemical difference or differing composition of the PM leaflets by introducing a spontaneous curvature term. The spontaneous curvature term $C_{o}$ accounts for this inhomogeneity with a value of zero for homogeneous leaflets and non‐zero otherwise. The classical continuum formulation for the bending energy proposed by Helfrich is

(2) $$E_{\text{Helfrich}}=\frac{k_{c}}{2}\int {\left(C-C_{o}\right)}^{2}\mathrm{dA}+k_{g}\int \left(C_{k}\right)\mathrm{dA}$$

where $k_{g}$ represents the Gaussian modulus and $C_{k}$ the Gaussian curvature where $C_{k}=C_{1}C_{2}$. The second term in Equation (2) is often omitted due to the Gauss–Bonnet theorem which provides a value for the integral based on the genus g, i.e., number of holes on the surface, such that $\int \left(C_{k}\right)\mathrm{dA}=4\pi \left(1-g\right)$. The genus of RBCs, or fully closed spherical topologies, is zero thus making the second term constant, i.e., 4π.

The other bending models are further extensions of the Canham and Helfrich models. The bilayer‐couple model was employed to simulate vesicles and RBCs [45] and it introduces the concept of area difference ΔA. The area difference is the difference in areas between the outside surface of the outer layer and the inside surface of the inner layer of the PM leaflets. In the bilayer‐couple model it is assumed that the PM leaflets maintain a constant area difference, and do not exchange lipid molecules; this leads to the implicit assumption that the PM leaflets are incompressible in this model [46]. The ADE model however is an extension of the bilayer‐couple model accounting for the additional bending energy due to changing area difference and is given by

(3) $$E_{\mathrm{ADE}}=\frac{k_{c}}{2}\int {\left(C-C_{o}\right)}^{2}\mathrm{dA}+\frac{{\bar{k}}_{c}}{2}\frac{\pi }{AD^{2}}{\left(\mathrm{\Delta A}-\Delta A_{o}\right)}^{2}$$

where ${\bar{k}}_{c}$ is the global bending rigidity, A is the area of the outer surface of the outer layer, D is the PM thickness, and ΔA and $\Delta A_{o}$ are the instantaneous and reference area differences, respectively. The reference $\Delta A_{o}$ is set as the desired value in this work.

The different bending models, given in Equations ((1), (2), (3)–(1), (2), (3)), calculate the bending energy based on the physiological state of the biomembrane. Regardless of this physiological state, the common term that contributes the most to the bending energy is

(4) $$E_{\text{bend}}=\frac{k_{c}}{2}\int {\left(C-C_{o}\right)}^{2}\mathrm{dA}$$

The formulation in Equation (4) is common throughout the literature with any additional terms accounting for the different physiological states of the PM; Equation (4) is the focus of the current work. In numerical RBC structural modelling, the treatment of curvature and the calculation of bending energy is very important. Continuum models enforce the effect of bending through force densities [47, 48, 49], i.e., local increases and decreases in forces due to bending effects or through the formulations shown in Equations ((1), (2), (3)–(1), (2), (3)). Discrete models on the other hand attempt to enforce the effect of bending energy through discrete formulations of the bending energy.

In discrete‐spring‐network structural models, the surface of interest is discretised into a mesh of contiguous, non‐overlapping triangles. The edges of the triangles are considered the springs and the connecting points of the springs are considered the nodes. This connection of springs and nodes is integral to the calculation of the curvature. Three forms of the bending energy for discrete‐spring‐network structural models, i.e., Equation (4), are presented next.

#### 2.1.1 BES A

The simplest form of bending energy used in the discrete‐spring‐network structural modelling of RBCs is from the formulation of Kantor and Nelson [36]. BES A is commonly seen in the literature [13, 49, 50] with the bending energy given as

(5) $$E_{\text{bend},A}=k_{b}\sum_{j=1}^{N_{s}}\left[1-\cos\left(\theta_{j}-\theta_{j,o}\right)\right]$$

**FIGURE 3:**
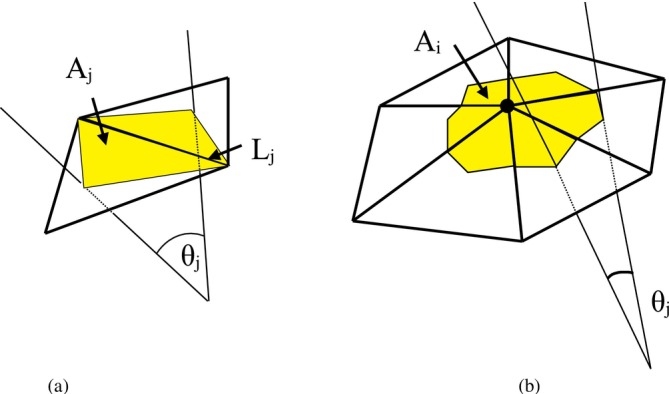
Bending on a triangulated spring‐network surface. $L_{j}$ represents the length of edge *j* in the spring network and $\theta_{j}$ is the angle subtended between the normals of the two neighbouring triangles sharing edge *j*. (a) Representative area $A_{j}$ used for the curvature calculation in BES B and (b) representative area $A_{i}$ used for the curvature calculation in BES C.

In the formulation for $E_{\text{bend},A}$, $k_{b}$ is the effective bending modulus, with $k_{b}=2\sqrt{3}k_{c}$ [51]; the calculation of the effective bending modulus can be seen in Appendix A. $\theta_{j}$ is the angle subtended between the normals of the two neighbouring mesh triangles sharing edge j (see Figure 3), $\theta_{j,o}$ is the reference angle subtended which is set as zero in this work, and $N_{s}$ is the number of triangle edges, i.e., springs, on the triangulated surface. $\theta_{j,o}$ is not equivalent to $C_{o}$ in Equation (4); a relationship can however be established through the spring edge length [44].

The value of $k_{b}=2\sqrt{3}k_{c}$ is based on the assumption of a sphere meshed with equilateral triangles only and $k_{b}$ should be recalculated for non‐spherical shapes or when any part of the mesh contains non‐equilateral triangles. This is not the case in the literature and an error is introduced into the definition of $k_{b}$ [52].

#### 2.1.2 BES B

BES B is adopted from the curvature definition proposed by Jülicher [37]. In this scheme, the physical bending modulus is not linked to a unique shape like BES A and the curvature is calculated directly based on spring elements. The curvature for any spring, $C_{j}$, is expressed as

(6) $$C_{j}=\frac{\theta_{j}L_{j}}{A_{j}}$$

and Equation (6) is substituted into Equation (4). The discretised bending energy is given as

(7) $$E_{\text{bend},B}=\frac{k_{c}}{2}\sum_{j=1}^{N_{s}}{\left(\frac{\theta_{j}L_{j}}{A_{j}}-\frac{\theta_{j,o}L_{j,o}}{A_{j,o}}\right)}^{2}A_{j}$$

where as shown in Figure 3, $L_{j}$ is the length of edge j, and $A_{j}$ is one‐third of the areas of the triangles sharing edge j. The subscript o represents a reference value which is set as the initial value for $L_{j,o}$ and $A_{j,o}$, whereas $\theta_{j,o}$ is set as zero as $C_{o}=0$ in this work. BES B is identical to the model ‘Jnoavg’ in the work of Tsubota [49]. It is shown explicitly in this work that BES B is equivalent to BES A under certain conditions as discussed in Appendix A.

#### 2.1.3 BES C

Other models exist that also include a direct calculation of the curvature. This direct calculation can be achieved either through the first moment of curvature or by calculating the Laplace–Beltrami operator. Gompper and Kroll [53] used the Laplace–Beltrami operator and the voronoi area at a node to calculate the curvature while Jülicher [37] used the first moment of curvature and the barycentric area at the node to calculate the curvature. Bian et al. [44] and Farnudi et al. [54] stated that the method of Jülicher [37] is more accurate and robust and is the method used in this work for direct curvature calculation. BES C differs from BES B as the curvature for each spring is averaged between the two nodes at either end of the spring, i.e., the curvature of each spring is divided by two. The curvature at node i is then given by

(8) $$C_{i}=\frac{\frac{1}{2}\sum_{j=1}^{N_{w}}\left(\theta_{j}L_{j}\right)}{A_{i}}$$

and

(9) $$E_{\text{bend},C}=\frac{k_{c}}{2}\sum_{i=1}^{N_{i}}{\left(\frac{\frac{1}{2}\sum_{j=1}^{N_{w}}\left(\theta_{j}L_{j}\right)}{A_{i}}-\frac{\frac{1}{2}\sum_{j=1}^{N_{w}}\left(\theta_{j,o}L_{j,o}\right)}{A_{i,o}}\right)}^{2}A_{i}$$

where $N_{w}$ is the number of springs meeting at node i, $N_{i}$ is the number of nodes, and $A_{i}$ is an area associated with node i and, as shown in Figure 3, is one‐third of the total area of the triangles that share node i.

### 2.2 Numerical Model Construction

In this section, the numerical methodology developed to predict the equilibrium shape, i.e., steady‐state shape, using either BES A, BES B or BES C for flat membranes, vesicles and RBCs is introduced. A spring‐network model of a RBC predicts its shape by conserving its volume and surface area while also accounting for shearing and bending resistance. This is achieved using the Helmholtz free energy, $E_{\text{total}}$, defined as

(10) $$E_{\text{total}}=E_{\text{volume}}+E_{\text{area}}+E_{\text{shear}}+E_{\text{bend}}$$

where $E_{\text{volume}}$, $E_{\text{area}}$, $E_{\text{shear}}$ and $E_{\text{bend}}$ are the respective component energies.

The cytosol volume constraint energy, $E_{\text{volume}}$, is

(11) $$E_{\text{volume}}=\frac{K_{V}}{2V_{o}}{\left(V-V_{o}\right)}^{2}$$

where $K_{V}$ is the volume‐constraint modulus, and V and $V_{o}$ are the instantaneous and reference volumes, respectively. The reference volume is the initial volume.

The RBC PM has a constant surface area; the area constraint energy, $E_{\text{area}}$, is given by

(12) $$E_{\text{area}}=\frac{K_{\mathrm{LA}}}{2}\sum_{k=1}^{N_{T}}\frac{{\left(A_{k}-A_{k,o}\right)}^{2}}{A_{k,o}}+\frac{K_{\mathrm{GA}}}{2A_{o}}{\left(A-A_{o}\right)}^{2}$$

The first part of Equation (12) accounts for the movement restriction due to the transmembrane and surface proteins attaching the cytoskeleton to the PM; it specifically serves to regularise the triangle areas. This is often referred to as the local area constraint energy where $K_{\mathrm{LA}}$ is the local area dilation modulus. Each triangular element on the surface of the discretised RBC has an index k, and $A_{k}$ and $A_{k,o}$ are the instantaneous and reference triangle areas, respectively. $N_{T}$ represents the total number of triangular mesh elements. The second part of Equation (12) is often referred to as the global area constraint energy as it enforces the surface incompressibility of the PM. $K_{\mathrm{GA}}$ is the global area dilation modulus, and A and $A_{o}$ are the instantaneous and reference surface areas, respectively. The reference triangle area is taken as the initial triangle area and the reference surface area is taken as the overall initial area.

The in‐plane shearing energy is due to the underlying cytoskeleton which accounts for the shearing resistance of the RBC; the worm‐like‐chain (WLC) spring is typically used in discrete‐spring‐network RBC models. It is adopted from the work of Marko and Siggia [55] who first used the WLC spring to study double‐stranded deoxyribonucleic acid (DNA) and Li et al. [8] who adopted the WLC spring to study RBCs. The shearing energy, $E_{\text{shear}}$, is given as

(13) $$E_{\text{shear}}=\frac{K_{B}T}{4p}\sum_{j=1}^{N_{s}}\left[L_{c,j}\left(\frac{3r_{j}^{2}-2r_{j}^{3}}{1-r_{j}}-4c_{1}r_{j}-c_{2}\right)\right]$$

where $K_{B}$ is the Boltzmann constant, T the absolute temperature, and p the persistent length, i.e., a mechanical property quantifying the stiffness. $L_{c,j}$ is the contour length of spring j. The ratio of instantaneous spring element length, $L_{j}$, to the contour length, $L_{c,j}$, is defined as $r_{j}$, i.e., $r_{j}=L_{j}/L_{c,j}$. The values of $c_{1}$ and $c_{2}$ are set as 0.7121 and −0.568, respectively, so that at the start of the simulation with $r_{j}$ set as 1/2.8, the spring has no shearing energy. For the WLC spring, the shear modulus $K_{S}$ is related to the spring network terms as follows:

(14) $$K_{S}=\frac{\sqrt{3}K_{B}T}{4{\mathrm{pL}}_{c,j}}\left[\frac{1}{2{\left(1-r_{j}\right)}^{3}}+1\right]$$

This equation is used together with the employed shear modulus value to calculate the value of the constant $K_{B}T/4p$ for each spring.

The PM accounts for resistance against bending. The bending energy is given in Equation (4). The continuum equation is substituted by one of the bending schemes, i.e., BES A, BES B or BES C.

The equilibrium shape of a model is determined via an iterative procedure to obtain converged nodal forces, i.e.,

(15) $$\left|f_{t,i}\right|<f_{m}$$

where for each node $f_{t,i}$ is the total nodal force consisting of the external loading force $f_{l,i}$ and the internal reaction force $f_{r,i}$, i.e.,

(16) $$f_{t,i}=f_{l,i}+f_{r,i}$$

and $f_{m}$ is the force convergence tolerance which is defined to be $1\times 10^{-5}$ of the maximum of all the initial forces $f_{t,i}$. The loading force $f_{l,i}$ is defined for each simulation as part of the loading conditions whereas the reaction force $f_{r,i}$ is calculated from the gradient of the model energy, i.e.,

(17) $$f_{r,i}=-\nabla \left[E\right]$$

In addition, the stiffness $K_{i}$ is calculated as the gradient of the reaction force, i.e.,

(18) $$K_{i}=-{\left.\nabla \left[f_{r,i}\right]\right|}_{E\to 0}$$

Once calculated, if all the nodal forces are smaller than the force tolerance $f_{m}$, convergence is deemed to have been achieved and the simulation stops. If the nodal forces have not converged, the nodal displacement ${\mathrm{ds}}_{i}$ is computed using

(19) $${\mathrm{ds}}_{i}=\frac{f_{t,i}}{K_{i}}$$

**FIGURE 4:**
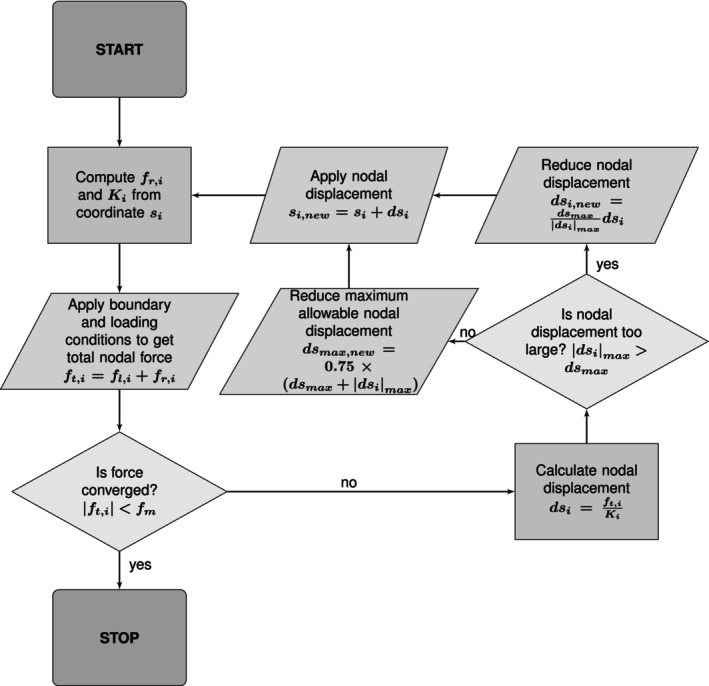
Flow chart showing the calculation of the structural equilibrium shape. $s_{i}$ is the nodal coordinate, $f_{r,i}$, $f_{l,i}$ and $f_{t,i}$ are the reaction, external loading and total nodal forces, respectively, $K_{i}$ is the stiffness, $f_{m}$ is the force convergence tolerance, ${\mathrm{ds}}_{i}$ is nodal displacement and $ds_{\max}$ is the maximum allowable nodal displacement.

To ensure numerical stability, the maximum nodal displacement calculated throughout the domain is restricted to a maximum allowable value which is initially defined to be $1\times 10^{-1}$ of the smallest edge length of the triangular mesh elements. If the maximum nodal displacement exceeds the maximum allowable value, all nodal displacements are reduced proportionally; otherwise they are allowed and the maximum allowable value itself is reduced. Once confirmed, the displacement is assigned to each node for another iteration. See the flow chart in Figure 4 for the iterative procedure for finding the equilibrium shape.

The developed numerical model allows for external forces to be applied through $f_{l,i}$. In this work only equilibrium steady‐state predictions are relevant and thus viscous effects are not considered, i.e., the model is a steady model.

## 3 Results and Discussion

In this section numerical predictions for flat membranes, vesicles and RBCs are presented in turn. First, an open flat membrane was examined under uniaxial bending where one end of the membrane was constrained while the other end experienced an out‐of‐plane bending force. The predictions are presented for all three bending schemes in conjunction with analytical results. Secondly, the flat membrane was subjected to isotropic bending with constrained corner nodes. The flat membrane testcases, i.e., Sections 3.1 and 3.2 are meant as illustrative benchmarks; for this reason, the material properties are set to clearly demonstrate the relative performance of the different schemes. The test cases represent the bending of a stiff, i.e., metal‐like, and a soft, i.e., foamy bubble‐like, material, respectively; the bending energy is large when bending a stiff material and small when bending a soft material.

Application to enclosed spring‐network membranes was also investigated. In these biologically relevant test cases, i.e., Sections 3.3 and 3.4, physiologically relevant material properties are used. Numerical predictions of equilibrium vesicles are first presented for the three bending schemes and compared against a vesicle phase diagram. Finally, equilibrium RBCs were numerically predicted using the three bending schemes and predicted shapes were compared against experimentally observed equilibrium RBC shapes.

### 3.1 Uniaxial Bending

#### 3.1.1 Problem Set‐Up

**FIGURE 5:**
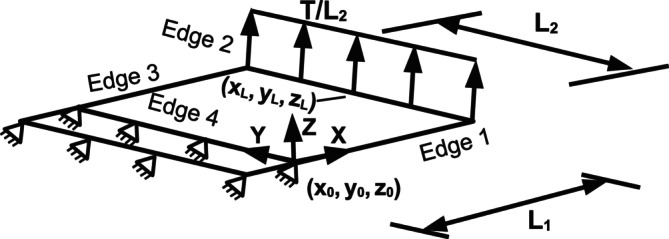
Boundary conditions for the uniaxial bending test case. The nodes along Edge 4 and on the attached strip were fixed while the nodes along Edge 2 were subjected to a uniformly distributed load $T/L_{2}$.

An initially flat membrane with dimensions $L_{1}=1\;m$ and $L_{2}=1\;m$ was subjected to out‐of‐plane bending in order to establish the differences between the three bending schemes. To enforce uniaxial bending, large values were set for the local area dilation and shear moduli in comparison to the bending modulus. The local area dilation modulus was set as $K_{\mathrm{LA}}=1\times 10^{7}\;N/m$, the global area dilation modulus as $K_{\mathrm{GA}}=0\;N/m$, the shear modulus as $K_{S}=1\times 10^{7}\;N/m$. The bending modulus was set as one unit solely for benchmarking, i.e., $k_{c}=1\;J$ and its scale does not affect the comparative trends of the different bending schemes. The large moduli values prevented changes in the membrane area and spring lengths thereby yielding no changes in the respective component energies, i.e., $E_{\text{area}}$ and $E_{\text{shear}}$; this is akin to bending a very stiff material. In order to easily compare predictions between different mesh densities, a strip with a dimension of one spring length × 1 m was attached to the membrane along Edge 4. All nodes on the strip were fixed while the nodes on Edge 2 were subjected to an evenly distributed loading force *T* in the *z* direction as shown in Figure 5. The force *T* was applied in steps of 0.1 *N* up to the final force of 2 *N* and uniformly distributed along Edge 2 as $T/L_{2}$.

An analytical solution to the uniaxial bending of the initially flat membrane is proposed in this work; it is proposed that the deformed geometry is given by

(20) $$z\left(x\right)=z_{L}\left(1-\cos\mathrm{ax}\right)$$

where a is a positive coefficient, and x, y and z are the coordinates in a right‐hand cartesian coordinate system as shown in Figure 5. $z_{L}$ is the upward deflection at point L. The analytical solution for the bending energy $E_{\text{bend},\text{analytical}}$ is

(21) $$E_{\text{bend},\text{analytical}}=\frac{k_{c}}{2}\int_{0}^{\frac{\pi }{2a}}{\left(\frac{a^{2}z_{L}\cos\mathrm{ax}}{{\left(1+a^{2}z_{L}^{2}\sin^{2}\mathrm{ax}\right)}^{\frac{3}{2}}}\right)}^{2}\mathrm{dx}$$

The full derivation of this solution is given in Appendix B.

#### 3.1.2 Results

**FIGURE 6:**
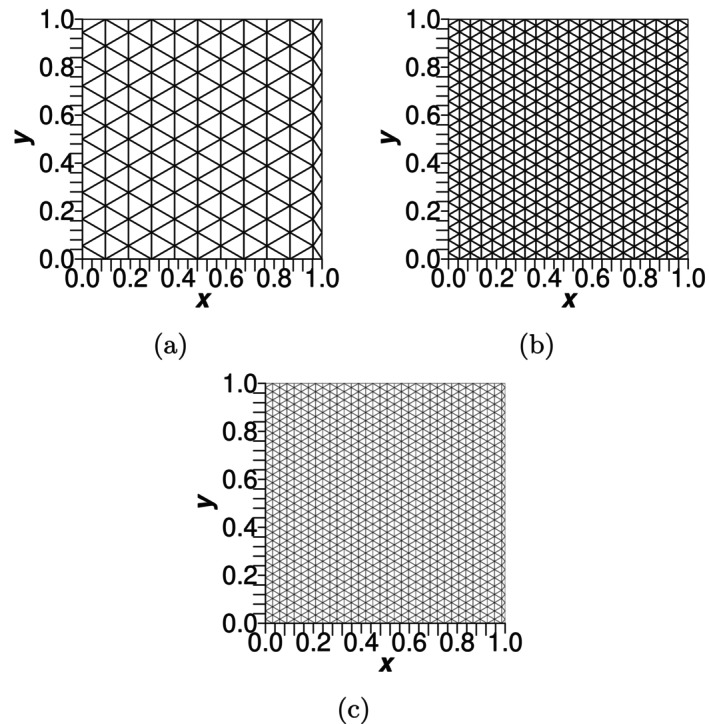
The spring‐network meshes used in the numerical simulations with (a) 136 nodes, (b) 492 nodes and (c) 1098 nodes.

**FIGURE 7:**
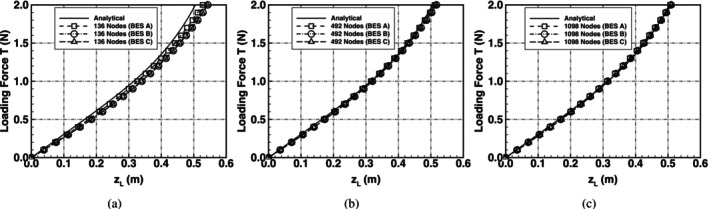
Comparison of predicted and analytical force–deflection curves of an initially flat spring‐network membrane undergoing uniaxial bending using meshes with (a) 136 nodes, (b) 492 nodes and (c) 1098 nodes.

The meshes used in the simulations are presented in Figure 6 followed by the predicted force–deflection curves in Figure 7. While it is common to see very high mesh densities in the literature, higher mesh densities are not required for the flat membrane test cases in this work as the model predictions showed good mesh convergence with low mesh densities.

Initial comparison using a mesh with 136 nodes shows that BES A, BES B and BES C required less loading force for the same level of deflection compared with the analytical solution. The differences in required loading force became less as the mesh density increased. For each scheme, mesh convergence was deemed to have been achieved when the deflection $z_{L}$ for a loading force T=2 N varied by less than 2% between meshes. Convergence for each of the three schemes was achieved with a mesh density of 1098 nodes.

**FIGURE 8:**
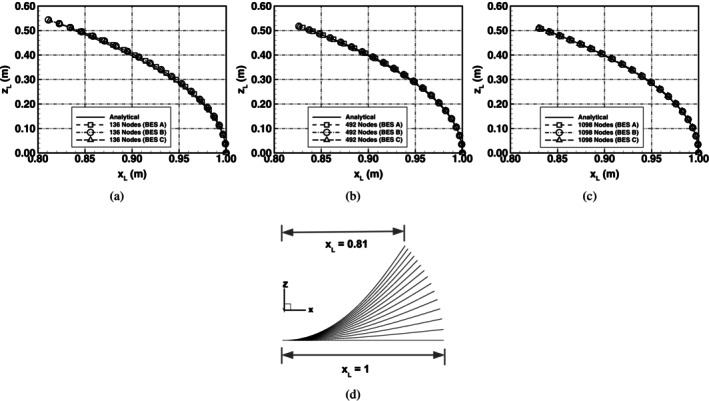
Deflection path of an initially flat spring‐network membrane undergoing uniaxial bending. (a) Comparison of the numerical predictions with the analytical solution using a mesh with 136 nodes, (b) 492 nodes, (c) 1098 nodes and (d) graphical representation of a sample bending path from initially flat, $x_{L}=1$, to the out‐of‐plane bent shape, $x_{L}=0.81$, using a mesh of 136 nodes and BES A.

The topology from the numerical predictions and the analytical solution matched up very well. The bending path, i.e., the path travelled by the loading edge is shown in Figure 8. The plot shows that there is very good agreement between the numerical bending schemes and the analytical solution showing that all the bending schemes accurately predicted the deformed geometry in uniaxial bending.

**FIGURE 9:**
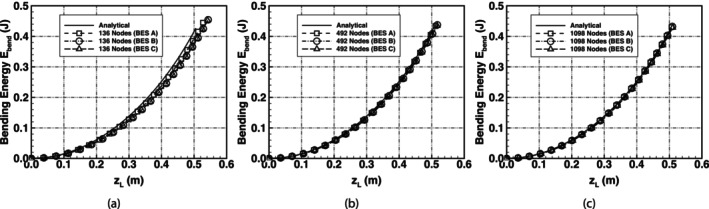
Comparison of predicted and analytical bending energy of an initially flat spring‐network membrane undergoing uniaxial bending using meshes with (a) 136 nodes, (b) 492 nodes and (c) 1098 nodes.

Using the proposed analytical bending energy solution in Equation (21), and the definition of the membrane bending energy in Equations (5), (7) and (9), a comparison was made between the analytical bending energy and the bending energy predicted using the numerical schemes; this comparison is shown in Figure 9. The results showed that there is very good agreement between the analytical solution and the predictions using the different bending schemes. The bending schemes can be used to accurately model cases of uniaxial bending where the bending mechanics do not include complex shape changes.

### 3.2 Isotropic Bending With Constrained Corner Nodes

#### 3.2.1 Problem Set‐Up

**FIGURE 10:**
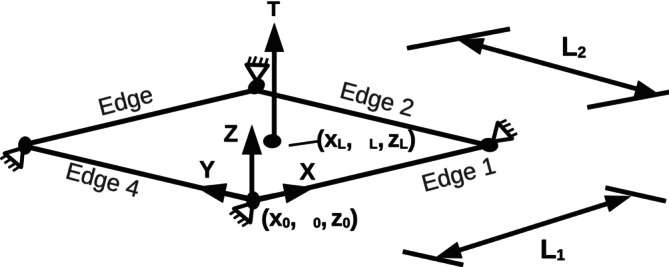
Boundary conditions for the isotropic bending test case with constrained corner nodes and mid‐point loading. The corner nodes were fixed while the mid‐point (point *L*) was subjected to an upward loading force *T* of 2 *N* (restricted to a maximum loading force which resulted in a maximum deflection $z_{L}=0.13\;m$).

This test case examined the bending profile of an initially flat membrane subjected to mid‐membrane loading and constrained corner nodes. As in the previous test case $L_{1}=1\;m$ and $L_{2}=1\;m$. The bending mechanics dominated the formation of the equilibrium shape as $K_{S}=0\;N/m$, $K_{\mathrm{LA}}=0\;N/m$, $K_{\mathrm{GA}}=0\;N/m$. Again the bending modulus was set as one unit, i.e., $k_{c}=1\;J$ and its scale does not affect the comparative trends of the different bending schemes. For the boundary conditions, shown in Figure 10, the corner nodes were fixed while the mid‐point (point *L*) was subjected to an upward loading force *T* of 2 *N* (restricted to a maximum loading force which resulted in a maximum deflection $z_{L}=0.13\;m$) in steps of 0.05 *N* for BES A and BES B, and steps of 0.001 *N* for BES C. The same mesh topology as employed in the previous test case was used albeit with the strip of one spring length × 1 m removed and mesh sizes of 85, 294, 2047 and 7965 nodes employed.

#### 3.2.2 Results

**FIGURE 11:**
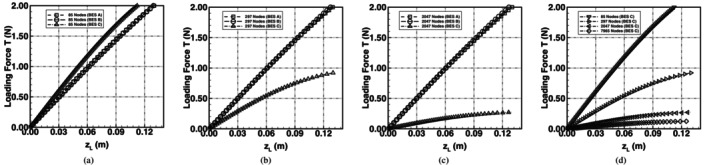
Comparison of force–deflection curves of an initially flat spring‐network membrane undergoing isotropic bending with constrained corner nodes and mid‐point loading. The predicted curves using BES A, BES B and BES C are shown using meshes with (a) 85 nodes, (b) 297 nodes and (c) 2047 nodes. The plot in (d) shows the force–deflection curves using BES C only, with an additional 7965‐node mesh introduced.

Plots of loading force *T* against the upward deflection of point *L*, i.e., $z_{L}$, are shown in Figure 11. The plots highlight the flexibility of the spring network using the different bending schemes. Using BES A and BES B the spring network was stiffer than BES C for the same deflection. BES C was very soft and significant shape change was predicted even with very small loads; the maximum deflection allowed was 0.13 m as the deflection increases significantly for further increases in the loading force.

**FIGURE 12:**
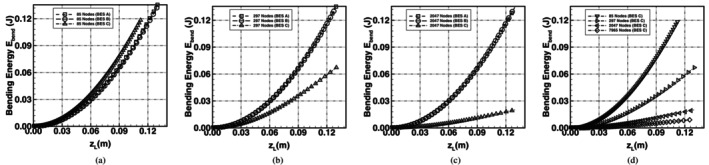
Comparison of the bending energy curves of an initially flat spring‐network membrane undergoing isotropic bending with constrained corner nodes and mid‐point loading. The predicted bending energy curves using BES A, BES B and BES C are shown using meshes with (a) 85 nodes, (b) 297 nodes and (c) 2047 nodes. The plot in (d) shows the bending energy curves using BES C only, with an additional 7965‐node mesh introduced.

**FIGURE 13:**
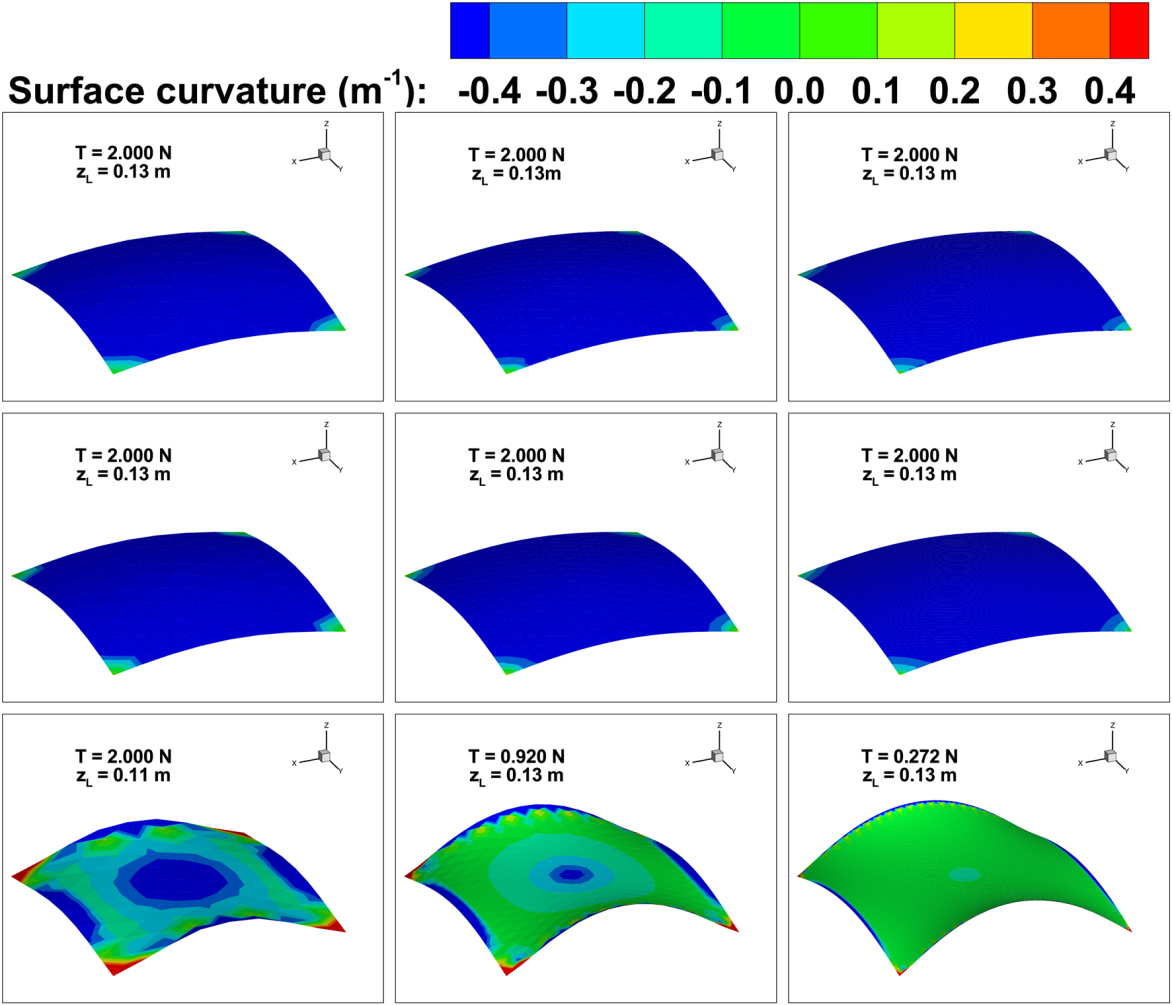
Isotropic bending of an initially flat spring‐network membrane with constrained corner nodes and mid‐point loading. Row 1 shows the predicted shapes using BES A for a spring network with 85 nodes, 297 nodes and 2047 nodes (left to right). Row 2 shows predictions using BES B while predictions using BES C are shown in Row 3. The predictions are for the last applied loading force for each scheme.

Plots of bending energy in Figure 12 show that BES C achieved the lowest bending energy when compared to BES A and BES B. The minimum energy increasingly trended towards zero with BES C only. The test case is analogous to the bending of an extremely soft material, i.e., foamy bubble‐like in nature, as it has no resistance to shearing. Under these conditions, an infinitesimal force produces a finite deformation of the membrane, and the associated bending energy is infinitesimal. As a result, the corresponding analytical solution in both Figures 11 and 12 is a straight horizontal line starting at the origin. Curvature plots are shown in Figure 13 and the curvature profile on the membrane with BES C is near zero, consistent with the analytical solution. The topology predicted with BES C was akin to a hyperbolic paraboloid saddle shape and, as the mesh was further refined, the surface curvature further approached zero. Using BES A and BES B, the curvature profile was non‐zero and the equilibrium shape was akin to a circular paraboloid.

The characteristics of BES C allow for significant shape change while BES A and BES B lead to a stiffer membrane with minimal shape change and bending. The inherent behaviour of the different bending schemes highlights that RBC shape changes driven by flow through blood vessels, or dictated by stomatocytogenic or echinocytogenic agents (which cause the stomatocyte‐to‐discocyte‐to‐echinocyte RBC transformation), may be more correctly captured in spring‐network RBC models by employing BES C. An additional test case for the bending of a soft material is presented in Data S1 where a flat membrane was subjected to bending with constrained mid‐side nodes.

### 3.3 Equilibrium Vesicle Shapes

#### 3.3.1 Problem Setup

A vesicle is basically a RBC without the cytoskeleton. Its wall structure is simple and any model for predicting this structure can be transferred to study more complicated cells like RBCs [44]. Different vesicle shapes exist based on the permeability of the wall leading to different cytosol volumes and area differences. This rich array of vesicle shapes presents an excellent test case to further understand the behaviour of the different bending schemes. In vesicle studies, vesicle shapes are often described with reference to a sphere with the same surface area as the vesicle leading to the introduction of the concepts of reduced volume and reduced area difference; the reduced volume, *v*, and the reduced area difference, Δa, are defined as

(22) $$v=\frac{V_{o}}{V}$$

(23) $$\Delta a=\frac{\Delta A_{o}}{8\mathrm{\pi RD}}$$

where *V* and *R* are the reference sphere volume and radius, respectively.

Using a numerical model, Seifert et al. [56] were able to propose a phase diagram of possible vesicle shapes at different reduced volumes, *v*, and reduced area differences, Δa; using BES A, BES B and BES C, vesicle shapes were predicted in this work and compared to the phase diagram, i.e., Figure 3 of Seifert et al. [56] to demonstrate the accuracy of the different bending schemes. The normalised bending energy predictions are compared to the results from Ziherl and Svetina [57]. To predict different vesicle shapes, the RBC network mechanics, i.e., Equation (10), was enforced without shearing resistance. The second term in Equation (3), i.e.,

(24) $$\frac{{\bar{k}}_{c}}{2}\frac{\pi }{{\mathrm{AD}}^{2}}{\left(\Delta A-\Delta A_{o}\right)}^{2}$$

was appended to Equations (5), (7) and (9) to drive the shape change. The continuum form of the total bending energy becomes $E_{\mathrm{ADE}}$ shown in Equation (3); it can be tuned through the choice of ${\bar{k}}_{c}$ to approximate the Helfrich model or the bilayer‐couple model [26, 46]. As ${\bar{k}}_{c}/k_{c}\to 0$, the energy converges to the Helfrich model, and as ${\bar{k}}_{c}/k_{c}\to \infty$, the energy converges to the bilayer‐couple model. Similar to the flat membrane test case, $C_{o}=0$. Using BES A, BES B, and BES C, vesicle shapes were predicted for a reduced volume, *v*, of 0.645 with varying Δa and predictions compared to the phase diagram of Seifert et al. [56] The starting equilibrium geometry was a discocyte with v=0.645 and Δa=1.1. The other model parameters are listed in Table 1.

**TABLE 1:** Model parameters for predicting different vesicle shapes at a reduced volume, *v*, of 0.645 and varying reduced area difference, Δa, values.

Model parameter	Value
$K_{V}$	$1000\;N/m^{2}$
$K_{\mathrm{LA}}$	100N/m
$K_{\mathrm{GA}}$	1000N/m
$K_{S}$	0N/m
$k_{c}$	$2.5\times 10^{-19}\;J$
${\bar{k}}_{c}$	$1\times 10^{-15}\;J$

#### 3.3.2 Results

**FIGURE 14:**
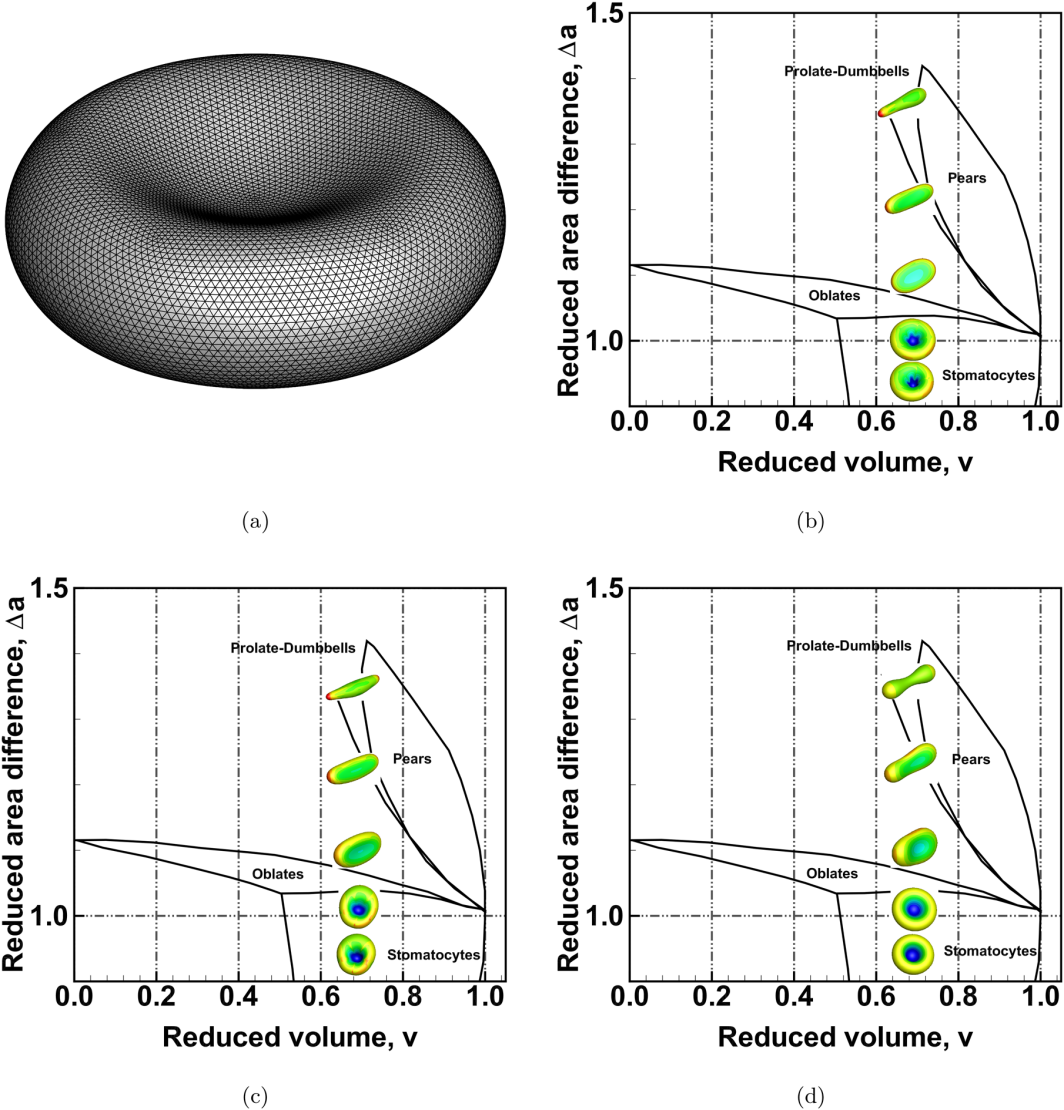
(a) Initial discocyte mesh with 10,242 nodes at a reduced volume, *v*, of 0.645 used in the numerical simulations. (b) Comparison of vesicle shapes at a reduced volume, *v*, of 0.645 for a reduced area difference, Δa, ranging from 0.92 to 1.35 using BES A. The predicted vesicle shapes are plotted against the phase diagram from Seifert et al. [56] (note that the colouring of the shapes relates to surface curvature). (c) Phase diagram comparison using BES B and (d) phase diagram comparison using BES C. The surface curvature legend is shown in Figure 15.

**FIGURE 15:**
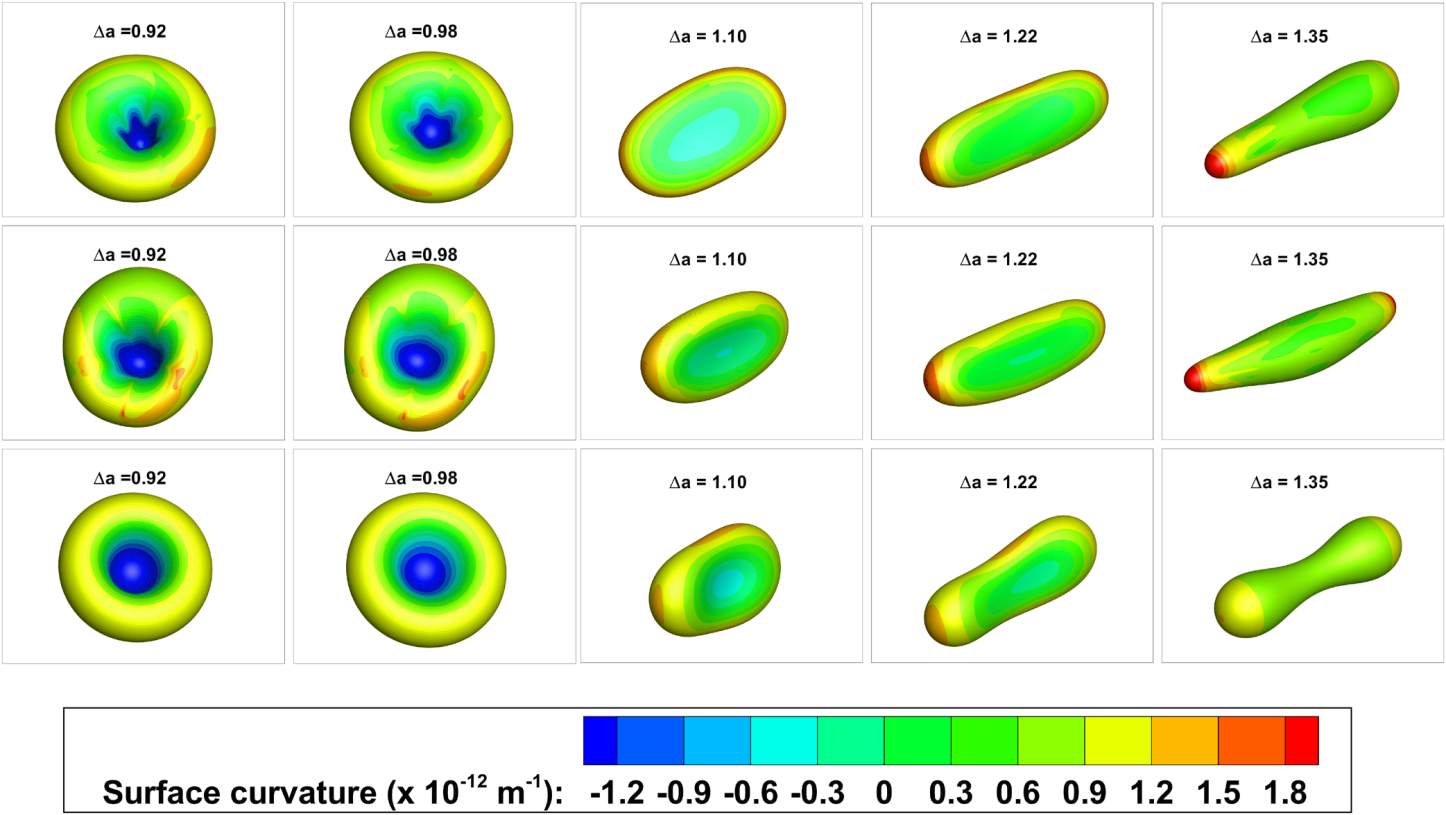
Predicted equilibrium vesicle shapes at a reduced volume, *v*, of 0.645 for a reduced area difference, Δa, ranging from 0.92 to 1.35 using BES A, BES B, and BES C. Row 1 shows the predicted shapes using BES A, Row 2 shows predictions using BES B while predictions using BES C are shown in Row 3.

The predicted vesicle shapes for all the bending schemes at a reduced volume, *v*, of 0.645 and varying Δa are shown in Figure 14 using a mesh with 10,242 nodes. This mesh density was deemed sufficient following a mesh convergence study. The reduced area difference, Δa, ranged from 0.92 to 1.35. All of the bending schemes predicted stomatocytic shapes for smaller Δa values. BES A and BES B were unable to predict any form of necking in the vesicle shapes. This became even more apparent with higher Δa values. In the diagram of Seifert et al. [56] the final vesicle shape, Δa=1.35, falls within the prolate‐dumbbell region. Only BES C correctly predicted a dumbbell shape with a pronounced neck; BES A and BES B were unable to predict this shape highlighting the deficiency in predicting more complex topologies with these bending schemes. Surface‐curvature‐coloured equilibrium vesicle shapes are shown in Figure 15. The plot shows more abrupt changes in surface curvature values with both BES A and BES B whereas the surface curvature with BES C has a smoother profile and a gradual change in the surface curvature values. The equilibrium shapes also vary distinctly with more axisymmetric shapes seen with BES C. The predictions using BES C also show a unique necking feature especially as the reduced area difference, Δa, increases; this necking feature does not appear with BES A and BES B.

**FIGURE 16:**
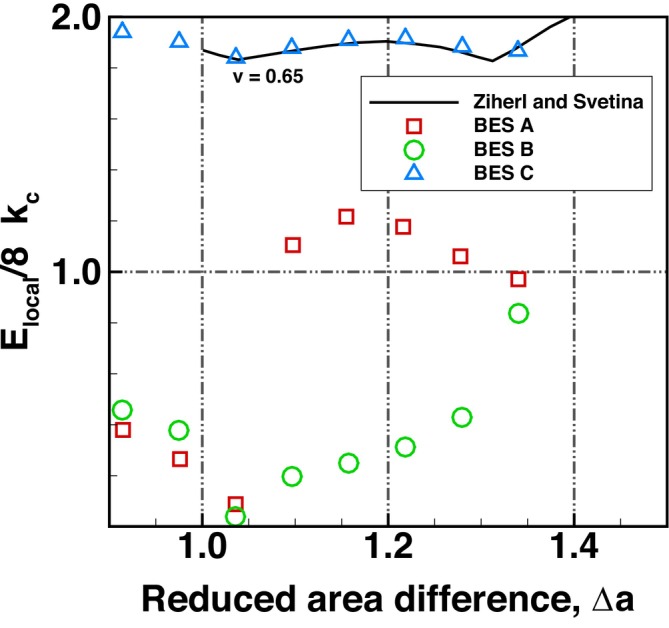
Comparison of the normalised bending energy of equilibrium vesicle shapes at a reduced volume, *v*, of 0.645 for a reduced area difference, Δa, ranging from 0.92 to 1.35 using BES A, BES B, and BES C plotted against results from Ziherl and Svetina [57].

A comparison of the normalised bending energy, $E_{\text{local}}/4\pi k_{c}$, is shown in Figure 16 for a range of Δa. $E_{\text{local}}$ is the predicted bending energy excluding the global energy (Equation 24). The predictions of BES A, BES B and BES C at v=0.645 were compared to results from Ziherl and Svetina [57] at v=0.65 (normalised bending energy data from Seifert et al. [56] are unavailable at this reduced volume v=0.645 and only cover a limited Δa range). As the predictions of BES A, BES B and BES C were at v≈0.65, BES C showed the best agreement to the results of Ziherl and Svetina [57], while BES A and BES B showed large deviations from the results of Ziherl and Svetina [57].

### 3.4 Equilibrium RBC Shapes

#### 3.4.1 Problem Set‐Up

Healthy RBCs have a discocytic shape; however, a range of other equilibrium RBC shapes exist due to physiological conditions in vivo like high or low pH levels, high or low salt levels, adenosine triphosphate depletion and diseased states or due to the presence of shape‐changing agents like cationic or anionic amphipathic drugs. These shapes typically span stomatocytes, where the RBCs contain a single concave indentation, to echinocytes, where the RBCs contain tiny spikes. The severity of the indentation or number of spikes serves as an identifier for the RBC name, e.g., echinocyte III contains more spikes than echinocyte I. The stomatocyte‐to‐discocyte‐to‐echinocyte (SDE) sequence is thought to occur as a result of variation in the reduced area difference, Δa. Lim et al. [23], using a continuum model, compared their numerical predictions of the SDE sequence with laboratory images ranging from stomatocyte III to echinocyte III. In this section, the same laboratory images from the work of Lim et al. [23] are presented and compared with the numerical predictions of the SDE transformation using BES A, BES B and BES C. The reduced area difference, Δa, was varied to drive the shape transformation and the reduced volume, *v*, was kept constant at 0.645. The model parameters employed in the test case are listed in Table 2 and compared with the parameters used in the work of Lim et al. [23] and reported experimental ranges.

**TABLE 2:** Comparison of model parameters for predicting different RBC shapes in the SDE sequence at a reduced volume, *v*, of 0.645 and varying reduced area difference, Δa, values against the model parameters used in the prediction of the SDE sequence from [23] and the reported range from experiments.

	Current work	Lim et al. [23]	Experimental range
Volume‐constraint modulus $K_{V}$ (N/m^2^)	1000	10,000	Not available
Local area dilation modulus $K_{\mathrm{LA}}$ (N/m)	100	—	Not available
Global area dilation modulus $K_{\mathrm{GA}}$ (N/m)	1000	1 × 10^−3^	Not available
Shear modulus $K_{S}$ (N/m)	4.5 × 10^−6^	2.5 × 10^−6^	(2–10) × 10^−6^
Bending modulus $k_{c}$ (J)	2.5 × 10^−19^	2 × 10^−19^	(2–9) × 10^−19^
Global bending rigidity ${\bar{k}}_{c}$ (J)	1 × 10^−15^	1.27 × 10^−19^	(1–6) × 10^−19^

#### 3.4.2 Results

A comparison of the numerical predictions in the SDE sequence using the different bending schemes is presented in Table 3. The results are presented for a mesh with 10,242 nodes. This mesh is the same as the mesh presented previously in the equilibrium vesicle shapes test case. The comparison shows that the shapes predicted using BES C qualitatively match the experimentally observed shapes for all points in the SDE sequence. The stomatocytes have a single concave indentation as seen in the experimentally observed images, and the spikes on the echinocytes are rounded with a necking feature. BES A and BES B on the other hand do not match up well with the experimentally observed shapes. The stomatocytes predicted using BES A have two concave indentations. The spikes predicted on the echinocytes using BES A are sharp and pointed and have no necking features. BES B predictions matched poorly with the experimentally observed images. The surface curvature predicted using BES B was irregular and varied significantly over the surface. The number of spikes counted on the predicted echinocyte I, II and III shapes is summarised in Table 4. These values are compared to the spike counts reported in experimental studies and previous numerical simulations. The spikes on the predicted shapes obtained with BES A and BES C fall within the range of reported values, with BES A generally yielding fewer spikes than BES C. The spike counts from BES B are not shown, as the predicted shapes did not correspond well to the experimentally observed shapes.

**TABLE 3:** Comparison of numerical predictions in the SDE sequence using BES A, BES B and BES C with experimentally observed shapes. The numerical predictions are for a reduced volume, *v*, of 0.645 and for a reduced area difference, Δa, ranging from 0.76 to 1.66. The experimentally observed images are reprinted with permission from [19, 58, 59].

Shape category	Δa	Experimentally observed	BES A	BES B	BES C
Stomatocyte III	0.76	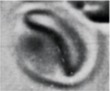	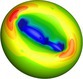	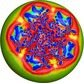	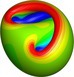
Stomatocyte II	0.83	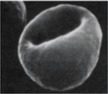	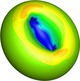	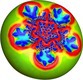	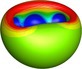
Stomatocyte I	0.98	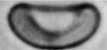	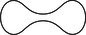	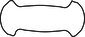	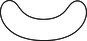
Discocyte	1.02	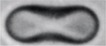	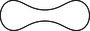	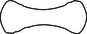	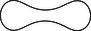
Echinocyte I	1.09	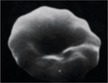	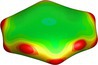	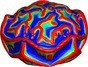	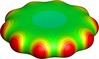
Echinocyte II	1.47	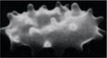	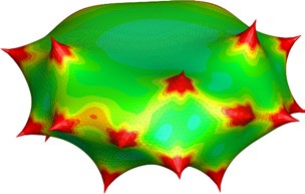	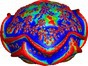	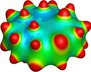
Echinocyte III	1.66	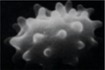	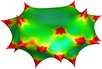	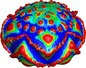	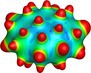
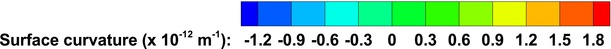					

**TABLE 4:** Number of spikes on predicted echinocyte shapes, compared with measured values reported from experimental studies [24, 26, 34, 60] and from prior numerical predictions [18, 24, 25, 26, 61].

	BES A	BES B	BES C	Measured values	Predicted values
Echinocyte I	8	—	10	—	6–12
Echinocyte II	17	—	22	—	15–23
Echinocyte III	20	—	31	10–50	30–41

**FIGURE 17:**
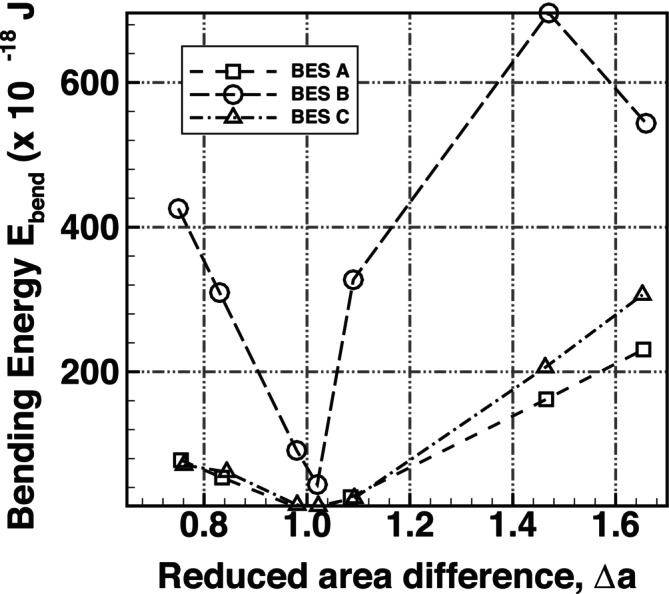
Comparison of the bending energy of predicted equilibrium RBC shapes at a reduced volume, *v*, of 0.645 for a reduced area difference, Δa, ranging from 0.76 to 1.66 using BES A, BES B, and BES C.

A comparison of the bending energy using the different bending schemes is presented in Figure 17. Surprisingly, the predicted bending energy matches up quite well between BES A and BES C, with a divergence in energy levels seen in the echinocyte II and III shapes. BES B has higher bending energy levels and appears to be stiffer than the other two schemes. In summary, the comparison between schemes highlights that BES C will yield more physiologically realistic predictions and is robust for predicting different equilibrium RBC shapes. BES A and BES B are limited to simple discocyte shapes and will yield physiologically unrealistic RBC shapes and bending mechanics.

## 4 Conclusions

This research has for the first time presented predictions from flat and enclosed spring‐network membrane test cases to demonstrate the effect of different treatments of the bending energy in discrete‐spring‐network structural models. The first flat membrane test case involving uniaxial bending, was akin to the bending of a very stiff material. The predictions showed that all three bending schemes performed equally well and closely matched the analytical results. The second flat membrane test case was akin to the bending of a very soft material. The predictions showed that BES A and BES B exhibited a stiff response while BES C on the other hand allowed for greater elasticity and deformation of the spring network. The analytical solution for loading force and bending energy was also better represented by BES C. An important note is that biological membranes, like vesicles and RBCs, are very soft materials. To confirm the phenomena seen in the flat membrane test cases, simulations involving enclosed membranes were conducted to compare predictions against known vesicle phase diagrams and complex RBC shapes. The enclosed membrane test cases again confirmed that BES C allowed for more elasticity and deformation of the spring network. Critically, BES C numerically reproduced the whole range of expected biological shapes. BES A and BES B on the other hand were unable to reproduce the vesicle phase diagrams and complex RBC shapes. In this regard, BES B performed very poorly in the last test case and the predicted shapes bore little resemblance to the biological RBC shapes or the predictions using the other bending schemes. This research also highlights that BES A, commonly used to simulate the bending mechanics of spring‐network membranes, has limitations and might underestimate the true bending deformation. As can be seen in the enclosed membrane test cases, the shape of the biological membrane might also be inaccurately predicted using BES A. To accurately capture complex RBC shapes, like echinocytes or rheological flow‐induced shapes, BES C should be used for the bending mechanics of RBC spring networks as it has proven to be accurate and robust in the shape predictions.

## Author Contributions

**Osayomwanbor Ehi‐Egharevba:** conceptualisation, formal analysis, investigation, methodology, software, visualisation, writing – original draft, writing – review and editing. **Mingzhu Chen:** conceptualisation, formal analysis, investigation, methodology, software, resources. **Fergal J. Boyle:** conceptualisation, funding acquisition, supervision, writing – review and editing.

## Ethics Statement

The authors have nothing to report.

## Conflicts of Interest

The authors declare no conflicts of interest.

## Supporting information


**Data S1:** Supporting Information.

## Figures and Tables

**FIGURE B1:**
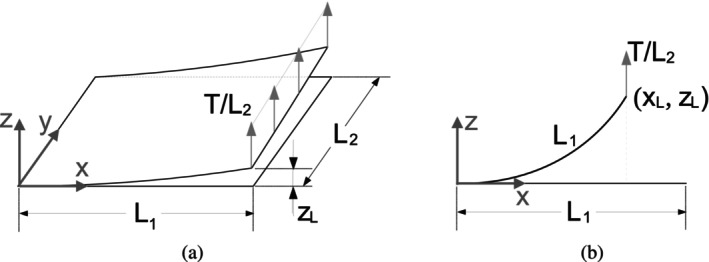
(a) An initially flat membrane undergoing out‐of‐plane bending due to a force T and (b) side view of the bent membrane in the x−z plane.

## Data Availability

The data that support the findings of this study are available within the article.

## Appendix A Derivation of the BES A Bending Modulus

The bending energy for BES A is based on the angle subtended between the normals to the two triangular mesh elements sharing the spring element (see Figure 3), with the total bending energy given by

(A1)
$$E_{\text{bend},A}=k_{b}\sum_{j=1}^{N_{s}}\left[1-\cos\left(\theta_{j}-\theta_{j,o}\right)\right]$$

where $k_{b}$ is the effective bending modulus, $\theta_{j}$ is the angle subtended between the normals of the two neighbouring mesh triangles sharing edge *j*, $\theta_{j,o}$ is the reference angle subtended, and $N_{s}$ is the total number of triangle edges. The bending force is the negative gradient of the bending energy and is

(A2)
$$F_{\text{bend},A}=-k_{b}\sum_{j=1}^{N_{s}}\left[\sin\left(\theta_{j}-\theta_{j,o}\right)\frac{d\theta_{j}}{\mathrm{ds}}\right]$$

The bending energy for BES B is

(A3)
$$E_{\text{bend},B}=\frac{k_{c}}{2}\sum_{j=1}^{N_{s}}\left[{\left(\frac{\theta_{j}L_{j}}{A_{j}}-\frac{\theta_{j,o}L_{j,o}}{A_{j,o}}\right)}^{2}A_{j}\right]$$

where $k_{c}$ is the physical bending modulus, $L_{j}$ is the length of edge *j*, and $A_{j}$ is one‐third of the areas of the two triangles sharing edge *j*. The subscript *o* represents a reference value. The bending force can be derived from the bending energy and is given by

(A4)
$$F_{\text{bend},B}=-\frac{k_{c}}{2}\sum_{j=1}^{N_{s}}\left[\left[2A_{j}\left(\frac{\theta_{j}L_{j}}{A_{j}}-\frac{\theta_{j,o}L_{j,o}}{A_{j,o}}\right)\frac{dC_{j}}{\mathrm{ds}}\right]+\left[{\left(\frac{\theta_{j}L_{j}}{A_{j}}-\frac{\theta_{j,o}L_{j,o}}{A_{j,o}}\right)}^{2}\frac{dA_{j}}{\mathrm{ds}}\right]\right]$$

Now, assuming that the triangular mesh elements do not undergo any area dilation or shearing, i.e., $A_{j}=A_{j,o}$ and $L_{j}=L_{j,o}$, then the following hold true

(A5)
$$\frac{{\mathrm{dA}}_{j}}{\mathrm{ds}}=0$$

and

(A6)
$$\frac{{\mathrm{dC}}_{j}}{\mathrm{ds}}=\frac{L_{j}}{A_{j}}\frac{d\theta_{j}}{\mathrm{ds}}$$

Under these conditions, the bending force for BES B reduces to

(A7)
$$F_{\text{bend},B}=-k_{c}\sum_{j=1}^{N_{s}}\left[\left(\frac{\theta_{j}L_{j}}{A_{j}}-\frac{\theta_{j,o}L_{j,o}}{A_{j,o}}\right)L_{j}\frac{d\theta_{j}}{\mathrm{ds}}\right]$$

or on re‐arranging

(A8)
$$F_{\text{bend},B}=-k_{c}\sum_{j=1}^{N_{s}}\left[\left(\theta_{j}-\theta_{j,o}\right)\frac{L_{j}^{2}}{A_{j}}\frac{d\theta_{j}}{\mathrm{ds}}\right]$$

If the deformation is small then

(A9)
$$\theta_{j}-\theta_{j,o}\approx \sin\left(\theta_{j}-\theta_{j,o}\right)$$

and, also, if

(A10)
$$k_{b}=k_{c}\frac{L_{j}^{2}}{A_{j}}$$

then Equation (A8) above further reduces to

(A11)
$$F_{\text{bend},B}=-k_{b}\sum_{j=1}^{N_{s}}\left[\sin\left(\theta_{j}-\theta_{j,o}\right)\frac{d\theta_{j}}{\mathrm{ds}}\right]$$

which is the same as Equation (A2) given earlier for BES A. Therefore, it can be seen that BES B is equivalent to BES A if the following hold true:

- No area dilation or shearing of the triangular mesh elements
- Small deformation
- $k_{b}$ and $k_{c}$ are related through Equation (A10).

Finally, if the triangular mesh elements are equilateral triangles, then

(A12)
$$A_{j}=\frac{\sqrt{3}}{4}L_{j}^{2}\frac{2}{3}=\frac{L_{j}^{2}}{2\sqrt{3}}$$

so that

(A13)
$$k_{b}=2\sqrt{3}k_{c}$$

## Appendix B Uniaxial Bending of A Flat Membrane

Consider the uniaxial bending of an initially flat membrane of size $L_{1}\times L_{2}$ which undergoes out‐of‐plane bending due to an applied force as shown in Figure B1. It is assumed in this derivation that $L_{1}=L_{2}=1$. It is proposed here that the geometry of the membrane can be given by

(B1)
$$z\left(x\right)=z_{L}\left(1-\cos\mathrm{ax}\right)$$

where *a* is a positive coefficient, and *x* and *z* are the coordinates in the horizontal and vertical directions, respectively. As shown in Figure B1, $\left(x_{L},z_{L}\right)$ is the coordinate of the membrane edge along which the force is applied and $x\in \left(0,1\right)$. It should be noted that

(B2)
$$a\;x_{L}=\frac{\pi }{2}$$

In this setup, the membrane lengths are not altered due to the applied force. Now the membrane length in the x−z plane can be calculated as

(B3)
$$\int \mathrm{dL}=\int_{0}^{x_{L}}\left(\sqrt{{\left(\frac{\mathrm{dz}}{\mathrm{dx}}\right)}^{2}+1}\right)\mathrm{dx}=\int_{0}^{\frac{\pi }{2a}}\left(\sqrt{a^{2}z_{L}^{2}\sin^{2}\mathrm{ax}+1}\right)\mathrm{dx}=1$$

Combining Equations (B2) and (B3), a given $x_{L}$ yields unique values of *a* and $z_{L}$, e.g., incrementally decrease $x_{L}$ from 1 to 0, each time calculating *a* using Equation (B2) and $z_{L}$ using Equation (B3). Thus different bent membrane geometries are obtained for different loading conditions using Equation (B1).

### Bending Energy

For the bent membrane, the bending energy, $E_{B}$, is given by

(B4)
$$E_{B}=\frac{k_{c}}{2}\int {\left(C-C_{o}\right)}^{2}\mathrm{dA}$$

where $k_{c}$ is the physical bending modulus, *C* and $C_{o}$ are the instantaneous and reference curvatures, respectively, and *A* is the surface area of the membrane. $C_{o}=0$ since the membrane is initially flat while the instantaneous curvature [62] can be calculated as

(B5)
$$C=\frac{\left|\frac{d^{2}z}{{\mathrm{dx}}^{2}}\right|}{{\left(1+\frac{\mathrm{dz}}{\mathrm{dx}}\right)}^{\frac{3}{2}}}$$

Differentiating Equation (B1) and substituting into Equation (B5) yields

(B6)
$$C=\frac{a^{2}z_{L}\cos\mathrm{ax}}{{\left(1+a^{2}z_{L}^{2}\sin^{2}\mathrm{ax}\right)}^{\frac{3}{2}}}$$

and substituting Equation (B6) into Equation (B4) gives

(B7)
$$E_{B}=\frac{k_{c}}{2}\int_{0}^{\frac{\pi }{2a}}\int_{0}^{1}{\left(\frac{a^{2}z_{L}\cos\mathrm{ax}}{{\left(1+a^{2}z_{L}^{2}\sin^{2}\mathrm{ax}\right)}^{\frac{3}{2}}}\right)}^{2}\text{dydx}$$

After completion of the *y* integration, and noting that $L_{2}=1$, the bending energy becomes

(B8)
$$E_{B}=\frac{k_{c}}{2}\int_{0}^{\frac{\pi }{2a}}{\left(\frac{a^{2}z_{L}\cos\mathrm{ax}}{{\left(1+a^{2}z_{L}^{2}\sin^{2}\mathrm{ax}\right)}^{\frac{3}{2}}}\right)}^{2}\mathrm{dx}$$

From the previously found *a* and $z_{L}$, for a particular $x_{L}$, the corresponding bending energy can be calculated via numerical methods, e.g., trapezoidal rule.

### Loading Force

In this setup, the deformation of the membrane is solely due to the upwardly applied load *T*. Therefore, the bending energy is also given by

(B9)
$$E_{B}=\int_{0}^{z_{L}}\int_{0}^{L_{2}}\frac{T}{L_{2}}\text{dydz}$$

As $\int_{0}^{L_{2}}T/L_{2}\mathrm{dy}=T$, where *T* is the loading force, the bending energy reduces to

(B10)
$$E_{B}=\int_{0}^{z_{L}}\mathrm{Tdz}$$

Using the calculated bending energy from Equation (B8), via the reverse trapezoidal rule the loading force *T* can be calculated.
