# Supplementary material for: Bending Energy Schemes for Discrete‐Spring‐Network Structural Modelling of Red Blood Cells
Source: Int J Numer Method Biomed Eng. 2025 Nov 19;41(11):e70114. doi: 10.1002/cnm.70114 (PMC12628744; doi:10.1002/cnm.70114)
Supplement: Supplementary file 1 — Data S1: Supporting Information. [file CNM-41-e70114-s001.pdf]

SUPPORTING INFORMATION

BENDING OF A FLAT MEMBRANE WITH CONSTRAINED MID-SIDE NODES

An initially-flat membrane of size $L_1 = 1\ m$ and $L_2 = 1\ m$ was subjected to out-of-plane bending for the three different bending energy schemes (BES). The model parameters were specified to minimise the effects of shear resistance and area constraint and to ensure bending effects dominated, i.e., $K_S = 0\ N/m$, $K_{LA} = 0\ N/m$, $K_{GA} = 0\ N/m$ and $k_c = 1\ J$. The boundary conditions applied are presented in Figure 1. The corner nodes along Edge 4 were fixed while the mid-side nodes on Edge 1 and Edge 3 were also fixed. On Edge 2, the midpoint node was subjected to a loading force T of 2 N (restricted to a maximum loading force which resulted in a maximum deflection $z_L = 0.35\ m$) in steps of 0.05 N with BES A and BES B, and in steps of 0.001 N with BES C.

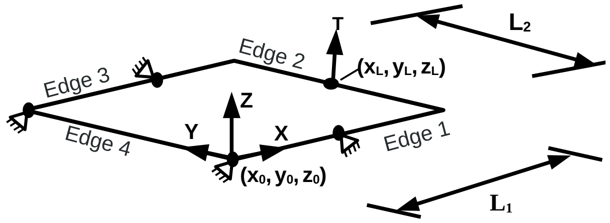

FIGURE 1 Boundary conditions for the bending test case with constrained mid-side nodes. The corner nodes on Edge 4 were fixed while the mid-side nodes on Edge 1 and Edge 3 were also fixed. On Edge 2, the midpoint (point L) was subjected to an upward loading force T of 2 N (restricted to a maximum loading force which resulted in a maximum deflection $z_L = 0.35\ m$).

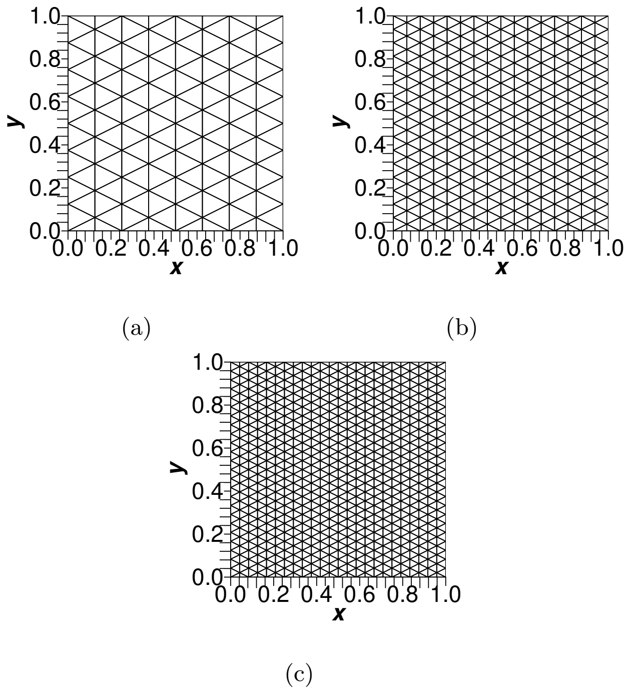

FIGURE 2 The spring-network meshes used in the numerical simulations with (a) 85 nodes, (b) 297 nodes and (c) 637 nodes.

Results

The meshes used in the simulations are shown in Figure 2. The test case represents the bending of an extremely soft material, i.e., foamy bubble-like in nature as there are no shearing effects. Under these conditions, an infinitesimal force produces a large deformation of the membrane and the associated bending energy is infinitesimal. Comparing the predicted force-deflection curves in Figure 3, it can be seen that the curves of BES A and BES B were stiffer than the curves of BES C. The membranes using BES C were very soft and large z_L deflections were predicted for very small loading forces. This matches the analytical solution which is a straight horizontal line starting at the origin of the plot in Figure 3. This means that numerical models using BES A and BES B might artificially reduce deflections caused by small loading forces.

A comparison of the bending energy in Figure 4 showed that the lowest bending energy was achieved with BES C; the predictions from this scheme better matched the analytical solution as the mesh density was refined. The analytical solution in this case is a straight horizontal line starting at the origin of the plot in Figure 4. Unlike the predictions using BES C, the predictions from BES A and BES B matched poorly to the analytical solution. The differences between the bending schemes can also be seen in the curvature plots shown in Figure 5. Based on the analytical solutions previously mentioned, a minimised surface curvature plot was expected. The results showed that the membrane topology using BES A and BES B were similar, however, curvature on the surfaces were not minimised. This is in contrast to the predictions from BES C where the membrane topology had a near-zero curvature profile.

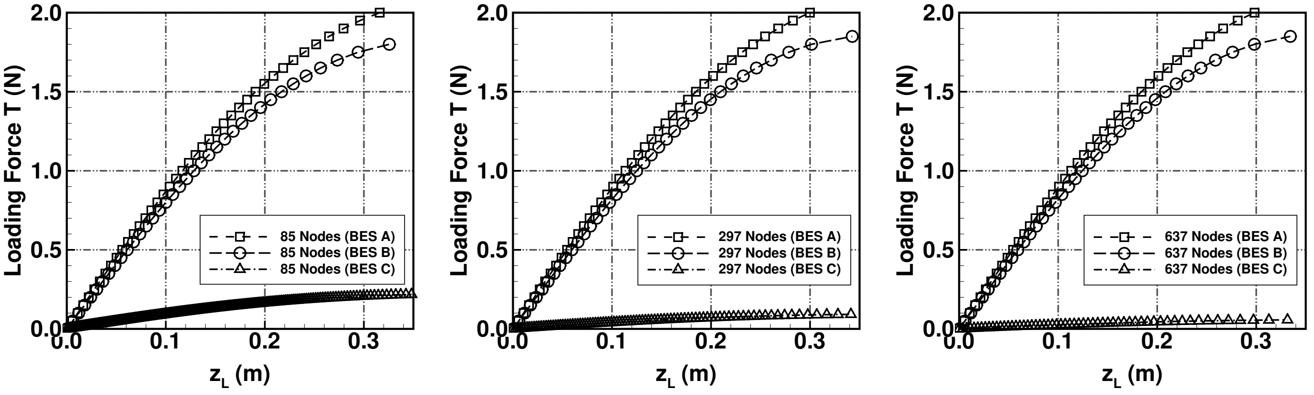

(a) (b) (c)

FIGURE 3 Force-deflection curves of an initially-flat spring-network membrane undergoing bending with constrained mid-side nodes. Predictions are compared using meshes with (a) 85 nodes, (b) 297 nodes and (c) 637 nodes.

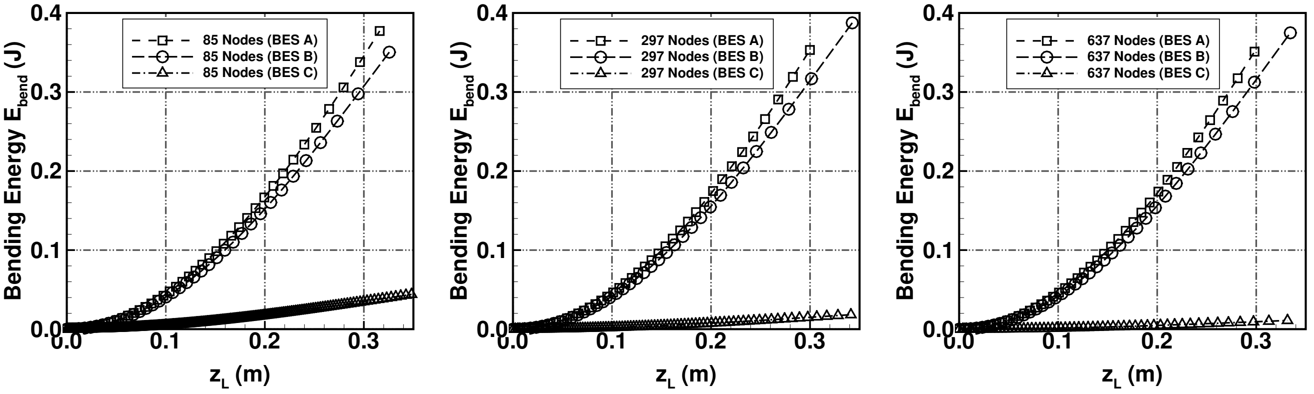

(a) (b) (c)

FIGURE 4 Comparison of the bending energy of an initially-flat spring-network membrane undergoing bending with constrained mid-side nodes. The predicted bending energy using BES A, BES B and BES C is shown using meshes with (a) 85 nodes, (b) 297 nodes and (c) 637 nodes.

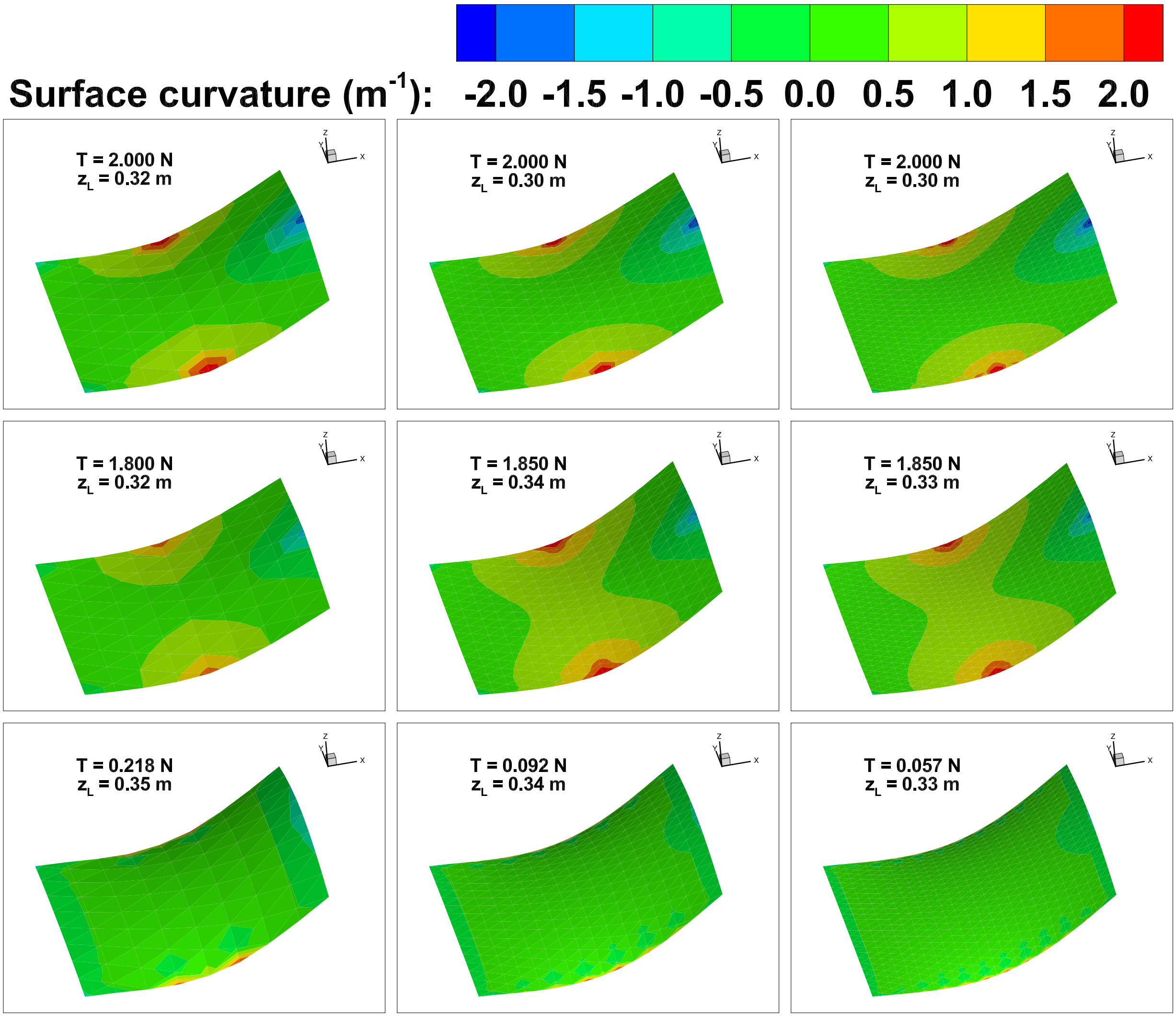

FIGURE 5 Bending of an initially-flat spring-network membrane with constrained mid-side nodes. Row 1 shows the results using BES A for a spring network with 85 nodes, 297 nodes and 637 nodes (left to right). Row 2 shows results using BES B while results for BES C are shown in Row 3. The results are shown for the last applied loading force for each scheme.
